# *In Vitro* and *In Vivo* Genotoxicity Assessment of *Aristolochia manshuriensis* Kom.

**DOI:** 10.1155/2012/412736

**Published:** 2012-07-11

**Authors:** Youn-Hwan Hwang, Taesoo Kim, Won-Kyung Cho, Hye Jin Yang, Dong Hoon Kwak, Hyunil Ha, Kwang Hoon Song, Jin Yeul Ma

**Affiliations:** KM-Based Herbal Drug Research Group, Korea Institute of Oriental Medicine, Daejeon 305-811, Republic of Korea

## Abstract

*Arisolochiae species *plants containing aristolochic acids I and II (AA I and AA II) are well known to cause aristolochic acid nephropathy (AAN). Recently, there are various approaches to use AAs-containing herbs after the removal of their toxic factors. However, there is little information about genotoxicity of *Arisolochiae manshuriensis* Kom. (AMK) *per se*. To obtain safety information for AMK, its genotoxicity was evaluated in accordance with OECD guideline. To evaluate genotoxicity of AMK, we tested bacterial reverse mutation assay, chromosomal aberration test, and micronucleus test. Here, we also determined the amounts of AA I and II in AMK (2.85 ± 0.08 and 0.50 ± 0.02 mg/g extract, resp.). In bacterial reverse mutation assay, AMK dose-dependently increased revertant colony numbers in TA98, TA100 and TA1537 regardless of metabolic activation. AMK increased the incidence of chromosomal aberration in Chinese hamster ovary-K1 cells, but there was no statistically significant difference. The incidences of micronucleus in bone marrow erythrocyte were significantly increased in mice after oral administration of AMK (5000 mg/kg), comparing with those of vehicle group (*P* < 0.05). The results of three standard tests suggest that the genotoxicity of AMK is directly related to the AAs contents in AMK.

## 1. Introduction

The stem of *Aristolchia manshuriensis* Kom. (AMK, *Gwanmoktong* in Korean, *Guanmuton* in Chinese and *Kanmokutsu* in Japanese) is harvested in many places of eastern Asia. For thousands of years, it has been traditionally used as a component of herbal medicine for the treatment of arthritis, rheumatism, hepatitis, pain relief, and diueresis due to their anti-inflammatory properties [1–3]. The traditional medicine literatures manifest AMK has ability of removal of heart fire, promotion of dieresis, restoration of menstruation, and enhancement of milk secretion [4, 5].

Aristolochic acids (AAs, I and II) are active ingredients of AMK. There was very little information on the toxicity of AAs-containing herbal medicine like AMK until nephrotoxicity and carcinogenicity of AAs-containing herbs were reported in Europe. Especially, in 1990s, nephrotoxicity of AAs-containing herbs had been firstly reported in the Belgian patients who had ingested *Stephania-tetrandra*-contained slimming pills [6]. After the substitution of *Aristolochiae species *for *S. tetrandra,* AAs-related nephrotoxicity, such as rapidly progressive interstitial nephritis, tubular necrosis, and end-stage renal diseases, was persistently induced. Similar cases of this nephropathy are called Chinese herb nephropathy (CHN) or aristolochic nephropathy (ANN) [4]. In China, a few cases of acute renal failure caused by an overdose of AMK were reported from 1964 to 1999 [7].

Several researchers have recently investigated the toxicity and side-effects of AAs-containing herbs in various species including human, mice, rat, and rabbit. Hu et al. [8] reported the acute and chronic toxicity of AMK in mice and rats after oral administration, which showed that the median lethal dose (LD_50_) of AMK was 29.2 ± 3.71 g/kg. They showed that AMK induced band-like necrosis in liver and tubular hydropic changes, interstitial inflammation, hyaline casts, and tubular regeneration in kidney. This nephrotoxicity was caused through the tubular cell apoptosis by AAs components contained in AMK [9, 10]. The no-observed-adverse-effect level (NOAEL) of AMK in mice was 0.06 g/kg/day, which is equivalent to 0.25 times of normal human dose in clinical prescription [11]. AAs also represented genotoxicity when it was tested using *in vitro* screening test including bacterial reverse mutation, mouse lymphoma cell gene mutation, and chromosomal aberration test [12]. They induced human urothelial cancer via their DNA-adduct property [13–15]. In humans, these adducts have been detected in kidney, ureter and urinary bladder, liver, and nontarget tissues such as pancreas, breast, and lung [14, 16]. Because of the increasing incidence of AAs-related nephrotoxicity and carcinogenicity, the therapeutic use of AMK and other AAs-containing herbs have been banned by government of United States of America, China, Japan, and Europe. In 2003, the Korean Food and Drug Administration (KFDA) had also banned AAs-containing medicinal herbs, including Radix *Aristolochiae* and Fructus *Aristolochiae*.

Recently, AAs-free herbs with similar therapeutic indication have been used in lieu of AAs-containing herbs. Most of AAs-free substitutes belong to different families [17]. The therapeutic equivalence of AAs-free substitutes may differ to those of AAs-containing herbs due to different taxonomy and action mechanism. Therefore, the removal of AAs from AAs-containing herbs can be a more practical approach to develop toxicity-free safe herbal medicine. Ling et al. [18] removed more than 80% of AAs (I and II) from AMK using a supercritical fluid extraction method. To remove AAs, they fermented AMK with fungi and mushroom and obtained more than 50% reduction of AAs of *Aristolochiae* species [19, 20]. In addition, Chung et al. [21] and Hegde et al. [22] found that novel phenanthrene compounds, such as aristopyridinone A, aristololamide II, and SCH546909, isolated from AMK have anti-inflammatory and antitumor activities. However, there was little information on their roles in mutagenicity and clastogenicity of AMK *per se*. In this respect, the establishment of genotoxic test methods and the characterization on the genotoxicity of AMK are inevitable for the therapeutic uses of AAs noncontaining AMK.

Therefore, this study was conducted not only to evaluate genetic toxicity of AMK on the basis of AAs contents but also to provide the adequate screening model on its genotoxicity. Three tests for characterizing mutagenicity of AMK were performed under Good Laboratory Practices (GLP) system: bacterial reverse mutation, chromosomal aberration, and micronucleus test, according to the guideline of Organization for Economic Cooperation and Development (OECD) 471, 473, and 474.

## 2. Materials and Methods

### 2.1. Chemicals and Reagents

HPLC grade methanol and acetonitrile (J. T. Bakers, Philipsburg, NJ, USA) were used for HPLC-DAD analysis. Furylfuramide (AF-2) was purchased from Wako (Osaka, Japan). Sodium azide (SA), 9-aminoacridine hydrochloride hydrate (9-AA), 2-aminoanthracene (2-AA), cyclophosphamide (CPA), mitomycin C (MMC), trifluoroacetic acid (TFA), aristolochic acid (AA) I, and AA I and II mixture were obtained from Sigma-Aldrich (St. Louis, MO, USA). Other chemicals were purchased from Sigma-Aldrich Co.

### 2.2. Preparation of AMK

The stem of AMK was collected from Yeongcheon, South Korea, in winter 2009. A voucher specimen was deposited in the herbarium of KM-Based Herbal Drug Research Group, Korea Institute of Oriental Medicine, registration number 350. Air-dried AMK (2.7 kg) were placed in 26 L of distilled water and then heated at 115°C for 3 h. The extracted mixture was filtered into standard sieves (150 *μ*m) (Restsch, Haan, Germany) and then lyophilized to yield crude extract. Brownish powder (260.4 g) was obtained and then stored at 4°C prior to use.

### 2.3. Quantification of AAs in AMK

**Figure 1:**
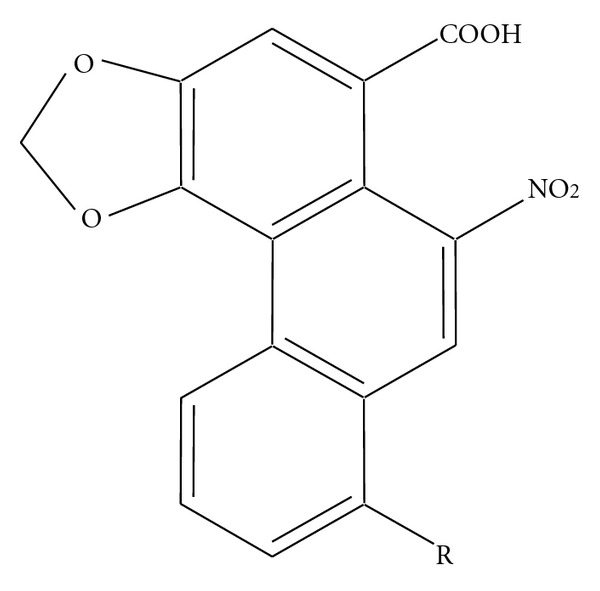
Chemical structure of aristolochic acid I (R=OCH_3_) and II (R=H).

To quantify AA I and AA II (Figure 1), standard stock solutions and AMK (10 mg) were dissolved in methanol and deionized water, respectively, and then filtered through a 0.45 *μ*m membrane filter before HPLC analysis. HPLC analysis was conducted on an Alliance 2695 instrument equipped with a PDA 996 detector (Waters Corporation, Milford, MA, USA). Data acquisition and analysis were performed by Empower 1 chromatography software (Waters Corporation). A C_18_ column (Phenomenex, Luna 5 *μ*m, 4.6 mm × 250 mm) with a guard cartridges column was used and maintained at 30°C. A gradient solvent composition of acetonitrile (A) and 0.1% TFA in deionized water was used as follows: 0–5 min, 10% B; 5–55 min, 10–85% B and finally washing column with 85% A for 10 min. The flow rate and injection volume were 1 mL/min and 20 *μ*L, respectively. The chromatograms were obtained at a wavelength of 240 nm. Standard stock solution (2.50–80.00 *μ*g/mL for AAI and 0.94–30.00 *μ*g/mL for AA II) was diluted in methanol. The calibration curves were plotted by peak area versus concentration of standard solution.

### 2.4. Metabolic Activation System

In the present study, S9 fraction induced by Aroclor 1254 was used for metabolic activation in *in vitro* assays. AAs are well-known activators for specific microsomal enzymes in liver and kidney, including cytochrome P450 (CYP) 1A1, CYP 1A2, NAD(P)H: quinone oxidoreductase (NQO1), and cyclooxygenase (COX) [23]. The metabolic activation system in *in vitro* genotoxic assays can be appropriately selected on the basis of characteristics of test compounds. Although the specific metabolic activation system for AAs may be used, we chose S9 fraction induced by Aroclor 1254 due to the genotoxic potentials of other components in AMK. In addition, the interpretation of results using S9 fraction induced by Aroclor 1254 may be useful for the comparison of those of other researchers [12, 24].

For the *in vitro* genotoxicity assays, rat liver S9 fraction induced by Aroclor 1254 was purchased from Moltox (Molecular Toxicology Inc., Boone, NC, USA). The S9 cofactor mix presents in the bacterial mutation assay consisted of 10% (v/v) S9 tissue fraction, 33 mM potassium chloride (KCl), 8 mM magnesium chloride (MgCl_2_), 4 mM nicotinamide adenine dinucleotide phosphate (NADP), 4 mM nicotinamide adenine dinucleotide (NAD), and 5 mM glucose-6-phosphate (G-6-P) prepared in 100 mM phosphate buffer (PBS, pH 7.4). For mammalian chromosomal aberration test, the S9 mix consisted of 30% (v/v) S9 tissue fraction, 5 mM MgCl_2_, 33 mM KCl, 5 mM G-6-P, 4 mM NADP, and 4 mM HEPES buffer prepared in the complete medium.

### 2.5. Bacterial Reverse Mutation Test

Plate incorporation and preincubation methods were conducted according to Maron and Ames [25] and OECD Guidelines for the Testing of Chemicals no. 471 [26]. The histidine-requiring *Salmonella typhimurium* TA98, TA100, TA102, TA1535, and TA1537, and the tryptophan-requiring *Escherichia coli *WP2*uvrA* were provided from Molecular Toxicology Inc. (Boone, NC, USA) and pre-incubated in Oxoid Nutrient Broth no. 2 at 37°C O/N. To determine an optimal range of AMK concentration, all strains were tested to AMK (dissolved in distilled water) in the presence and absence of a metabolic activation system (rat liver S9 mix). Based on the results of solubility and cytotoxicity evaluation, a range of AMK concentration (313–5000 *μ*g/plate) was selected for the main study. Following the plate incorporation method, 0.1 mL of bacterial suspension (Oxoid Nutrient Broth No. 2), 0.05 mL of test substance (AMK, vehicle and positive mutagens), and 0.5 mL S9 mix or PBS buffer (pH 7.4) were added to 2 mL of top agar (containing 0.6% agar, 0.5% NaCl, and 10% histidine/biotin or tryptophan solution). In the preparation of top agar, 0.5 mM of L-histidine/biotin for *S. typhimurium* strains and 0.5 mM tryptophan for *E. coli* strain were used. The mixture was preincubated for 20 min at 37°C and then poured onto a minimal glucose agar plates (1.5% agar, 1% Vogel-Bonner medium E, and 2% glucose). After incubation for approximately 48 h at 37°C, his+ and trp+ revertant colonies were counted. In the presence or absence of metabolic activation, each concentration of test substance was conducted triplicate in two independent experiments. The reference mutagens used as positive controls in each experiment without metabolic activation were as followings; AF-2 for TA98, TA100, and WP2*uvrA*, SA for TA1535, and 9-AA for TA1537. Different concentrations of 2-AA for TA98, TA100, TA1535, TA1537, and WP2*uvrA* were used with metabolic activation. Positive controls of each bacterial strain in the presence and absence of metabolic activation are summarized in Table 1. The test substance was considered positive in bacterial reverse mutation assay when there is (a) an increase (≥twofold number) of spontaneous revertants comparing with those of negative control or (b) a dose-dependent increase of revertant colonies in at least one of the tester strains without cytotoxicity.

### 2.6. Chromosomal Aberration Test of AMK in Chinese Hamster Ovarian Cells

The chromosome aberration study was conducted in accordance with OECD Guidelines for the Testing of Chemicals no. 473 [27] and Ishidate [28]. Chinese hamster ovary (CHO) K1 cells were obtained from American Type Culture Collection (ATCC, Manassas, VA, USA). The cells were cultured in F-12 Nutrient Mixture (Gibco BRL, Grand Island, NY, USA), supplemented with 10% fetal bovine serum (Hyclon Laboratories, Logan, UT, USA). Subculture was performed every 3-4 days to prevent overgrowth.

To determine the highest concentration of AMK for the main study, a dose-range finding study was performed with or without metabolic activation. The relative cell count was determined by comparing the cells numbers in AMK (39.06–5000 *μ*g/mL) and vehicle control cultures. In dose-range finding study, AMK (2500 mg/mL) after 6 h and 22 h treatment exhibited less than 50% of cytotoxicity in the absence of S9 activation, whereas 5000 mg/mL of AMK induced cytotoxicity approximately 40% of cytotoxicity at 6 h treatment in the presence of S9 fraction. Thus, the dose range of AMK for the main study was designed to consider its solubility and cytotoxicity in Table 2. MMC (0.04 *μ*g/mL) and cyclophosphamide (10 *μ*g/mL) were used as positive controls without or with the S9 fraction, respectively. Distilled water was used as the vehicle control. AMK and positive controls were diluted in distilled water. The CHO-K1 cells were seeded at 4 × 10^4^ cells/plate and incubated overnight. After preincubation, the cells were treated with AMK for 6 h with or without S9 fraction and for 24 h without S9 fraction. The cells at the end of treatment were washed with Ca^++^- and Mg^++^-free Dulbecco's phosphate buffered saline and fresh media was added. Colchicine 0.2 *μ*g/mL (Colcemide, Gibco, BRL, Grand Island, NY, USA) was added to each culture approximately 22 h after the initial treatment and further incubated during 2 h. All treatments were duplicated at each concentration. After incubation, slides of CHO-K1 cells for metaphase plate analysis were prepared after fixation with acetic acid:methanol (1 : 3, v/v) for 3-4 h and stained with 5% Giemsa solution (Merck, Darmstadt, Germany) for 5 min. At least 100 well-spread metaphase cells per slide were analyzed and the chromosome aberration were counted and recorded [29]. The data was statistically analyzed with the Chi-square test using SPSS 12.1 program (SPSS Inc. Chicago, IL, USA).

### 2.7. Bone Marrow Micronucleus Test of AMK in Mice

Micronucleus test was performed in compliance with Schmid [30] and OECD Guidelines for the Testing of Chemicals no. 474 [31]. Animal experiments were carried out after the approval of the Institutional Animal Care and Use Committee of Korea Conformity Laboratories (KCL). Thirty 7-week-old male ICR mice (27–30 g) were obtained from Orient Bio Inc. (Seongnam, South Korea). The animals were maintained in polycarbonate cage (*n* = 3). All animals were acclimated under laboratory condition after at least 1 week. Feed pellets and tap water after filtration were provided *ad libertum*.

In the dose-range finding study, oral administration of AMK did not induce any adverse effect such as death, clinical symptom, and decrease of bone marrow proliferation at a dose of 5000 mg/kg up to 48 h after treatment. For the main study, the animals were administered with a single oral dose of 0, 1250, 2500, and 5000 mg/kg body weight (10 mL/kg). AMK was dissolved in distilled water (D.W.) and D.W. served as a vehicle control. MMC was injected intraperitoneally at a dose of 2 mg/kg as a positive control. All animals were observed for general health state and body weight was measured before bone marrow sampling. At the end of experiment, mice were sacrificed by cervical dislocation and the femurs were obtained for bone marrow from surviving animal in each group. The bone marrow flushed using fetal bovine serum. After spreading and air-drying bone marrow cells on slides, the slides were fixed in methanol, stained 4% Giemsa solution and acridine orange (40 *μ*g/mL), and protected by mounted coverslip.

According to Hayashi et al. [32], the slides of bone marrow were observed for micronuclei and counted the ratio of polychromatic erythrocytes (PCE) and normochromatic erythrocytes (NCE) using light and fluorescence microscope. Two hundred erythrocytes and 2000 PCE per animal were scored to determine the ratio of PCE/NCE and the incidence of micronucleated polychromatic erythrocytes (MNPCE), respectively. One-way analysis of variance (ANOVA) using SPSS 12.1 program was used to evaluate the statistical significance between AMK-treated group and control group.

## 3. Results and Discussion

AMK, an AAs-containing herbal medicine harvested from eastern Asia, had been worldwidely used for the treatment of arthritis, rheumatism, hepatitis, and obesity. However, the pharmaceutical use of AAs-containing herbal medicines including AMK was banned in many centuries in the 2000s, as AAs were confirmed as potent carcinogens and mutagens in animals and human. To ensure the therapeutic equivalence of AMK substituent, many researchers recently attempted to use AMK after the removal of AAs. However, there was little information on the toxicity of AMK *per se*. In the present study, we quantified the contents of AA I and II in AMK and then confirmed the mutagenicity and clastogenicity of AMK on the basis of the contents of AA I and II. To evaluate the genotoxicity of AMK, we used the standard battery of assays including bacterial reverse mutation assay in five strains of *S. typhimurium* and *E. coli*, chromosomal aberration assay in CHO-K1, and micronuclei assay in mice bone marrow, which are recommended by the International Conference on Harmonization of Technical Requirements for Registration of Pharmaceuticals for Human Use (ICH).

### 3.1. Quantitation of AA I and II in AMK

**Figure 2:**
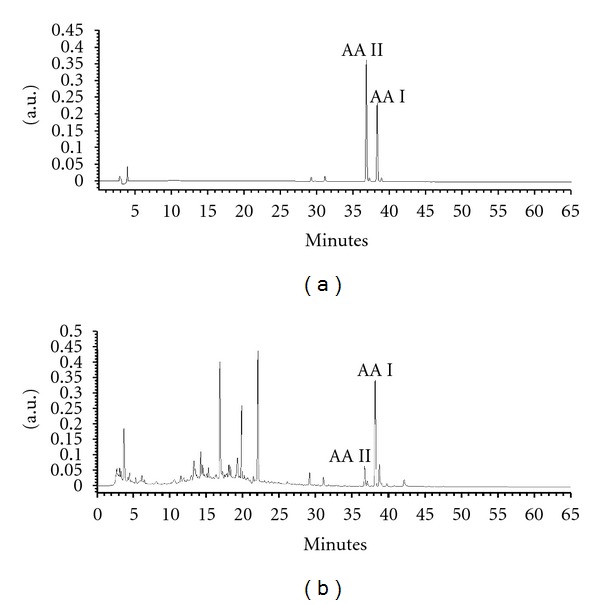
HPLC chromatogram of *Aristolochiae manshuriensis* Kom. (AMK) at 240 nm. (a) Aristolochic acid I (AA I, 38.263 min) and aristolochic acid II (AA II, 36.804 min); (b) amount of AA I (2.85 ± 0.08 mg/g extract) and AA II (0.50 ± 0.02 mg/g extract) in AMK.

A Phenomenex Luna C_18_ column and gradient elution with 0.1% TFA and acetonitrile were contributed to the good separation of AA I and II. The retention times of AA I and II were 38.263 min and 36.804 min, respectively (Figure 2). The linear regression equation were *y* = 101670*x* ± 68300 (*r* ^2^ = 0.9996) for AA I and *y* = 106676*x* ± 27116 (*r* ^2^ = 0.9991) for AA II. The limit of quantitation was 2.5 *μ*g/mL for AA I and 0.94 *μ*g/mL for AA II. The calibration curve was in a sufficient range to apply that to determine the amounts of AA I and II contained in AMK. We found that the amounts of AA I and II contained in AMK extract were 2.85 ± 0.08 mg/g and 0.50 ± 0.02 mg/g, respectively.

### 3.2. Bacterial Reverse Mutation Test of AMK

**Figure 3:**
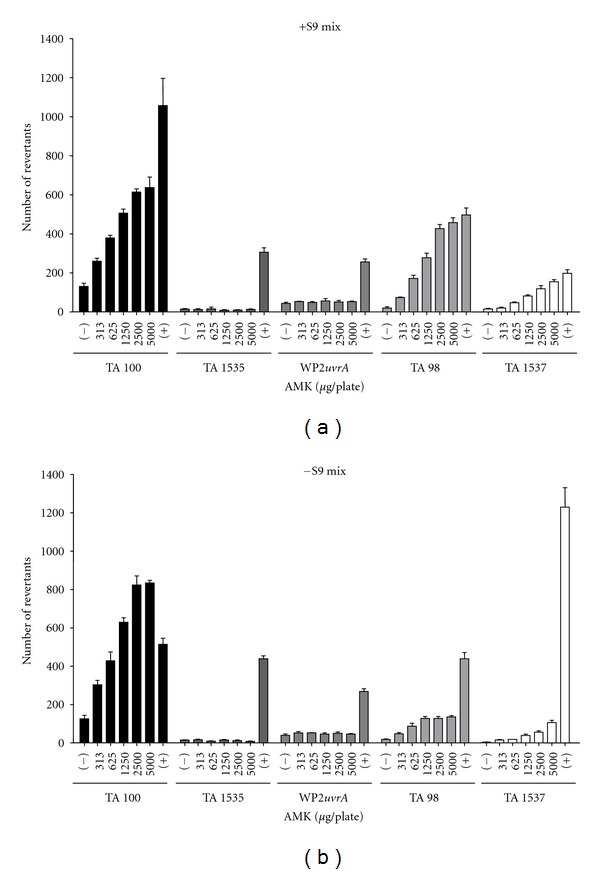
Effect of *Aristolochiae manshuriensis* Kom. (AMK) on bacterial reverse mutation in the presence (+S9) or absence (−S9) of rat liver S9 mix. (−), vehicle control; (+), positive control.

Bacterial reverse mutation assay is an initial *in vitro* screening method to evaluate potential genotoxicity of herbal substances and preparation [33]. This assay is performed to reveal the mutagenic potential of a substance and its reactive metabolites in a prokaryote organism with or without metabolic activation. In the present study, the mutagenicity of AMK in the presence or absence of S9 mix was assessed up to maximal concentration of 5000 *μ*g/plate using the histidine or tryptophan auxotroph bacteria strains, because there was no antibacterial activity on the test strains at any dose in the dose-range finding study. Regardless of S9 mix, AMK (313–5000 *μ*g/plate), which is equivalent to AAs (0.84–14.25 *μ*g/plate for AA I and 0.16–2.5 *μ*g/plate for AA II), caused dose-dependent increases of bacterial revertants in TA98, TA100, and TA1537 (Figure 3). But, there was no significant difference between AMK-treated and vehicle control in TA1535 and WP2*uvrA*. The positive controls significantly induced the mutation frequencies, verifying the sensitivity of the strains on mutagen. These results are consistent with previous reports [12, 34].

Robisch et al. [35] reported that AA mixture induced reverse mutation in TA100 and TA1537. In addition, AA I and II (1–1000 *μ*g/plate) were mutagenic to TA98 in the presence or absence of metabolic activation [12, 24, 36]. In particular, AMK induced base-pair substitution mutations (TA100) and frameshift mutations (TA98 and TA1537). TA98 and TA100 are more sensitive than their counterparts TA1537 and TA1535 because of their presence in pKM101 plasmid [25]. Likewise, TA98 and TA100 among the positive tester strains were more sensitive than TA1537 in this study. These results indicate that for rapid screening of AMK, preparation the prior use of TA98 and TA100 could be recommended in the respect with shorter time-consuming and lower cost. Taken together, AMK has a potent mutagenicity, and these three tester strains (TA98, TA100, and TA1537) were very sensitive to mutagen and useful for AMK-induced genotoxicity test using bacterial reverse mutation assay.

### 3.3. Chromosomal Aberration of AMK in CHO-K1 Cells

The *in vitro* chromosome aberration assay is usually used to determine and characterize the clastogenicity of testing substances that could induce structural chromosome aberrations in cultured mammalian cells. CHO-K1 cells are very sensitive to mutagen and have accumulated information for evaluation test of mutagenic and/or carcinogenic agents. In this study, we assessed whether AMK (625–5000 *μ*g/mL, equivalent to 2.0–16.75 *μ*g AAs/mL) causes the structural and numerical aberration in CHO-K1 cells. The results of chromosomal aberration by AMK are summarized in Table 2. The highest concentration (5000 *μ*g/mL) of AMK showed >50% of relative cell count (RCC) in the preliminary study. With or without metabolic activation system, the incidences of structural and numerical chromosomal aberrations did not show statistical difference between AMK-treated and negative control. But AMK nonsignificantly increased the chromosomal aberration in relation with the reduction of RCC values. In *in vitro* chromosomal aberration assay, cytotoxicity induction by nonmutagens could cause false-positive results. From that reason, positive responses by non-specific action are not relevant to human risk [37, 38]. Many researchers reported a positive role of AAs on chromosomal damage in various cell systems including human lymphocyte, mouse lymphoma cell, and CHO [12, 24, 39]. Especially, Zhang et al. [12] demonstrated that AAs significantly induced dose-related chromosomal damages in both mouse lymphoma and CHO. In this study, however, chromosomal aberration damages by AMK (2.0–16.75 *μ*g AAs/mL) did not have statistical differences in comparison with those of vehicle control, although the chromosomal aberration by AMK was observed dose dependently. These results are consistent with those of Zhang et al. [12]. They demonstrated that remarkable increases of chromosomal aberration at high concentration (≥25 *μ*g/mL). Besides, other components contained in AMK, to have antimutagenic property or to disturb the clastogenic effects of AAs in AMK have to be concerned as a reason of mutagenicity. The results indicate that CHO-K1 chromosomal aberration assay could be constrained to detect clastogenicity of AMK in spite of a very sensitive system.

### 3.4. Bone Marrow Micronucleus Test of AMK in Mice

**Figure 4:**
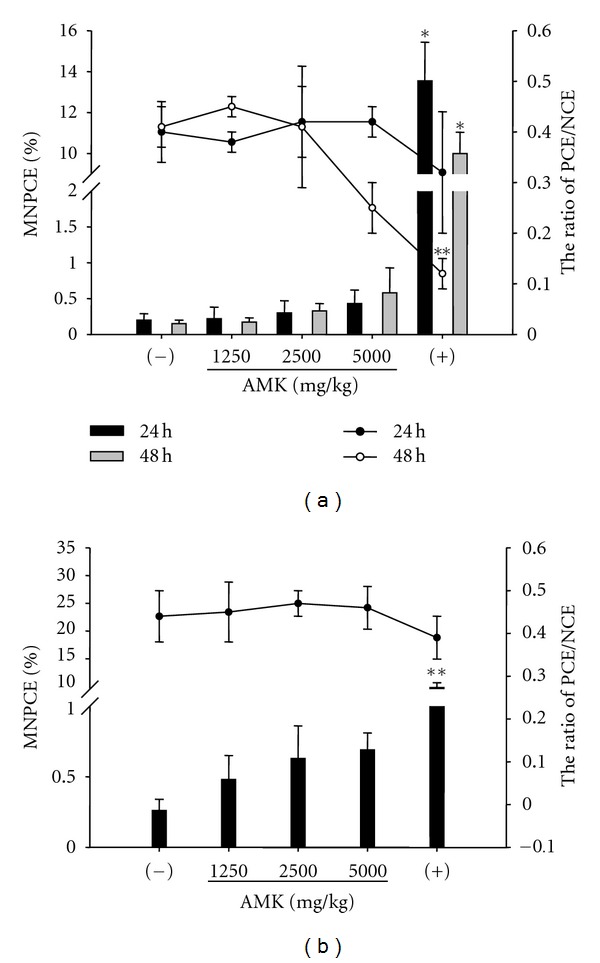
Effect of *Aristolochiae manshuriensis* Kom. (AMK, 0, 1250, 2500, and 5000 mg/kg body weight, oral administration) on the incidence (%) of micronucleated polychromatic erythrocyte (MNPCE, histogram bar, left *Y*-axis) and the PCE/NCE (polychromatic/normochromatic erythrocyte) ratio (curve with dot, right *Y*-axis) in preliminary ((a), *n* = 3) and main study ((b), *n* = 6). (−), vehicle control; (+), positive control (mitomycin C, MMC, 2 mg/kg). Asterisks denote statistically differences (*P < 0.05, **P < 0.01).

*In vivo* micronucleus assay, which determine substance-related chromosomal or mitotic damages in peripheral blood cells or bone marrow cells, is a useful tool to overcome the limitation of *in vitro* system and to provide more valuable information with consideration of affecting factors for genotoxicity. According to OECD guideline, three dose levels should be used in dose-range finding test and covered a range from the maximum to little or no toxicity [31]. The highest dose may be defined as a dose that produces some indication of toxicity on the bone marrow. Mengs and Klein [40] reported that AAs induced MNPCE in mice after intravenous administration (6 mg/kg). In this study, the highest dose (5000 mg/kg) of AMK (AA I, 2.85 ± 0.08 mg/g extract; AA II, 0.50 ± 0.02 mg/g extract) was selected due to the oral absorption of AAs and maximum dose of its preparation. We found that there were no clinical signs and behavior changes for all experimental period.  Figure 4 shows the incidences of micronuclei formation and the ratio of PCE/NCE in mouse bone marrow after single oral administration of AMK (1250, 2500, and 5000 mg/kg). In dose-range finding study, AMK induced dose- and time-dependent increases in MNPCE frequency after AMK administration, compared to the vehicle control (Figure 4(a)). The MNPCE frequency was higher in the 48 h samples than in the 24 h samples. The MNPCE of AMK at the highest dose (5000 mg/kg) in main study was approximately 3 times higher than of the control (*P* < 0.05, Figure 4(b)). AMK (5000 mg/kg) and MMC (2 mg/kg) significantly reduced the ratio of PCE/NCE at 48 h after AMK administration, indicating bone marrow suppression and cytotoxicity (*P* < 0.01, Figure 4(a)). In this respect, when we checked the PCE and NCE at 24 h after treatment in the main study, AMK did not significantly affect on the ratio of PCE/NCE (Figure 4(b)).

There was no available information for the clastogenicity of AMK *per se *in *in vivo* micronucleus assay. Mengs and Klein [40] demonstrated that a single intravenous injection of AAs (6, 20 and 60 mg/kg) significantly increased the numbers of micronucleated erythrocyte over negative control in mouse bone marrow micronucleus assay. Kohara et al. [41] and Chen [24] reported that AAs did not induce micronucleus formation in peripheral blood erythrocyte after oral administration (15 mg/kg/week during 4 weeks). With respect to a route of clinical administration (per oral), in this study, AMK (1250–5000 mg/kg, equivalent to 4.2–16.75 mg AAs/kg) dose dependently increased the incidence of MNPCE without induction of the significant difference of PCE/NCE ratio (Figure 4). Taken together, our results suggest that AMK is a potent clastogen *in vivo* and mouse bone marrow micronucleus assay confirmed that AMK has a genotoxicity, consistent with the results in the bacterial reverse mutation assay.

## 4. Conclusion

Recently, the uses of AAs-containing herbal extract after the removals of AAs are focused because of the critical problems about the pharmacological equivalence of AAs-containing herb substituent. This investigation was designed to quickly detect a genotoxicity of AMK using a set of screening tests including bacterial reverse mutation, chromosomal aberration, and micronucleus assay, recommended by ICH. In conclusion, the mutagenicity and clastogenicity of AMK in accordance with the contents of AA I and II were confirmed via bacterial reverse mutation assay and micronucleus assay, and these assays are very useful systems to determine the genotoxicity of AMK extract candidates prior to future clinical applications. Although mouse bone marrow micronucleus assay is a very helpful system for the determination of AMK genotoxicity, further study like repeated dose-micronucleus assay could be considered because of the very low concentration of AAs exposure after the removals of AAs.

## Figures and Tables

**Table 1:** Chemicals used as positive controls for each tester strain with or without metabolic activation system (rat liver S9 mix).

Strain	Without S9 mix		With S9 mix	
	Positive control^a^	Dose (*μ*g/plate)	Positive control^a^	Dose (*μ*g/plate)
*Salmonella typhimurium*				
TA100	AF-2	0.01	2-AA	1.0
TA1535	SA	0.5	2-AA	2.0
TA98	AF-2	0.1	2-AA	0.5
TA1537	9-AA	80.0	2-AA	2.0
*Escherichia coli*				
WP2*uvrA *	AF-2	0.01	2-AA	10.0

^a^AF-2: Furylfuramide; SA: sodium azide; 9-AA: 9-aminoacridine hydrochloride hydrate; 2-AA: 2-aminoanthracene.

**Table 2:** Effects of *Arisolochiae manshuriensis* Kom. (AMK) on the chromosomal aberration in Chinese hamster ovary (CHO)-K1 cells.

Treatment (*μ*g/mL)		AA I/ II (*μ*g/mL)	S9 mix	Time (h)^a^	Aberrant metaphases (−Gap/+Gap)	PP + ER	RCC^b^ (%)
Vehicle control		−	−	6–18	0.0/0.0	0.0	100.0

	625	1.78/0.31	−	6–18	1.0/1.5	0.0	86.5
AMK	1,250	3.56/0.63	−	6–18	0.5/0.5	0.0	77.8
	2,500	7.13/1.25	−	6–18	2.0/2.0	0.0	54.6

MMC	0.04	−	−	6–18	22.0/22.5^∗^	0.0	−
						0.0	

Vehicle control		−	+	6–18	0.5/0.5	0.0	100.00

	1,250	3.56/0.63	+	6–18	0.5/0.5	0.0	92.1
AMK	2,500	7.13/1.25	+	6–18	0.5/1.0	0.0	86.2
	5,000	14.25/2.50	+	6–18	1.0/1.5	0.0	69.1

CPA	10.00	−	+	6–18	27.0/27.5^∗^	0.0	−
						0.0	

Vehicle control		−	−	24–0	0.5/0.5	0.0	100.0

	625	1.78/0.31	−	24–0	0.5/1.0	0.0	75.1
AMK	1,250	3.56/0.63	−	24–0	1.5/2.0	0.0	72.5
	2,500	7.13/1.25	−	24–0	2.5/2.5	0.0	53.9

MMC	0.04	−	−	24–0	27.0/28.0^∗^	0.0	−

Abbreviations: PP: polyploidy; ER: endoreduplication; RCC: relative cell counts; MMC: mitomycin C; CPA: cyclophosphamide.

^a^Treatment time-recovery time.

^b^RCC (relative cell count) equals (no. of treated cells/no. of control cells) × 100 (%).

^∗^Significantly different from the vehicle control at *P* < 0.05.
